# Sensory Evaluation of Vanillin Obtained by Fungi in the Solid-State Fermentation from Agri-Food Industry By-Products

**DOI:** 10.3390/molecules30204109

**Published:** 2025-10-16

**Authors:** Ewa Szczepańska, Jacek Łyczko, Teresa Olejniczak

**Affiliations:** Department of Food Chemistry and Biocatalysis, Wrocław University of Environmental and Life Sciences, Norwida 25, 50-375 Wroclaw, Poland; jacek.lyczko@upwr.edu.pl (J.Ł.); teresa.olejniczak@upwr.edu.pl (T.O.)

**Keywords:** vanillin, SSF, fungi, biosynthesis, by-products, sensory evaluation, sensory panel

## Abstract

Vanillin is the compound widely used in the food industry as a flavoring agent. Currently, chemically synthesized vanillin provides the majority of the world’s supply. Due to the increase in consumer awareness, there is a change in preferences towards natural food additives. The main goal of this research was to obtain vanillin through Solid-State Fermentation on agri-food by-products such as brewer’s spent grain, wheat bran, and linseed oil cake. A specially designed SSF culture single-use bag bioreactor made of a poliamide-6 foil sleeve was used to conduct the process on a bench-scale (600 g of dry medium). After extraction and purification, obtained vanillin samples were subjected to sensory analysis to determine whether the origin of microbiologically obtained vanillin affects its aromatic properties. The panelists assessed that the extracts obtained from the cultures of *P. chrysosporium* CBS246.84 and *F. culmorum* MUT5855 proved to be attractive flavors as they showed more attractive sensory properties than synthetic vanillin and were comparable to commercially available vanilla bean extract. This is the first study to include sensory analysis of vanillin obtained biotechnologically by the SSF method.

## 1. Introduction

Vanillin (4-hydroxy-3-methoxybenzaldehyde) is an organic chemical compound, primarily known as the main component of vanilla flavor. It occurs naturally in pods of tropical vanilla plant *Vanilla planifolia* [1]. Vanillin is widely used as a flavoring and fragrance ingredient in the food and cosmetic industries. In the global market, around 1% of vanillin is derived from vanilla pods, while the majority is synthetic and sourced from lignin [2]. The price of natural vanillin is significantly higher than that of its synthetic counterpart, primarily due to the limited supply of vanilla plants. Obtaining this compound from natural resources is time-consuming, labor-intensive, and is highly dependent on natural factors such as climate change and plant diseases [3]. Synthetic vanillin is used to meet the vast majority of the global demand for vanilla flavoring (around 85%). The most common method today involves a two-step process using petrochemical precursors like guaiacol and glyoxylic acid [4]. In response to growing consumer preference for natural and sustainable products, there is a growing focus on reducing the use of compounds obtained through chemical processes and substituting them with more natural alternatives [5]. The bio-vanillin market was valued at 290.7 million USD in 2024 and is projected to hit a market valuation of 454.9 million by 2033 (CAGR of 5.1%). A 2024 survey of over 10,000 participants revealed that 7 out of 10 consumers are more likely to buy products with a “natural flavor” claim (https://www.astuteanalytica.com/industry-report/bio-vanillin-market, accessed on 25 July 2025). The European Union’s Regulation (EC) No. 1334/2008 states that the vanillin obtained by microbial transformations of natural precursors is considered as “natural flavor” [6]. Therefore, a range of biotechnological methods have been suggested, as researchers concentrate on microbial fermentation as a more environmentally sustainable and economically viable strategy for vanillin production. The biotechnological production of vanillin involves utilizing various microorganisms, such as bacteria and fungi, along with genetically modified microbes. This process allows for the bioconversion of compounds like ferulic acid, eugenol, isoeugenol, aromatic amino acids, glucose, and plant stilbenes [7,8,9,10,11,12,13,14].

Within this framework, utilizing lignocellulose by-products, a plentiful renewable resource, as a feedstock for vanillin production presents a compelling alternative. Lignin is the second most prevalent biopolymer on the planet, making up 15–30% of the mass of plant cell walls [6]. One of the compounds primarily found in lignin is ferulic acid; it plays a significant role in cell wall structure and function [15]. Due to structural similarities, ferulic acid stands out as one of the most widely utilized precursors for the biosynthesis of vanillin. The natural sources of this phenolic compound encompass waste and by-products from the agri-food sector, including sugar beet pulp, rice and oat husks, wheat straw, wheat bran, corn cobs, pistachio shells, coconut coir, and pomegranate and pineapple peels. Therefore, the attempts of introducing ferulic acid released from this lignocellulosic waste into microbiological cultures as a substrate were made, resulting in receiving vanillin as a product. The use of various microorganisms for this purpose was extensively studied using bacteria like *Streptomyces sannanensis*, *Streptomyces* sp., *Pediococcus acidilactici*, *Amycolatopsis thermoflava*, *Enterobacter hormaechei*, *Bacillus aryabhattai,* and fungi like *Aspergillus niger* and *Pycnoporus cinnabarinus* [7,8,13,16,17,18,19,20,21]. Solid-State Fermentation (SSF) has emerged as a new biotechnological method for biosynthesis of this compound [22]. The process is conducted mainly by fungi, as it mimics their natural habitat. Moreover, fungal strains are characterized by their ability to secreting lignin-modifying enzymes that are essential for the breakdown and modification of lignin leading to the formation of high-value compounds, including vanillin [2,23,24,25]. This culture system was used utilizing agri-food by-products such as rice straw, brewer’s spent grains, and coconut husk by *Serpula*, *Phanerocate*, *Aspergillus*, and *Fusarium* strains, which resulted in vanillin production [26,27,28]. SSF offers several advantages over submerged fermentation (SmF), such as environmental (provides a sustainable way to manage waste, significantly reduces water consumption and wastewater generation) and economic (uses cheap agricultural and industrial by-products as substrates, requires less energy for agitation and aeration compared to the large, stirred bioreactors used in SmF) [29]. Moreover, the low moisture content in SSF makes it less prone to contamination by bacteria, which typically thrive in high-water-activity environments [30].

Microbiological and lignin-derived vanillin can have a distinct and sometimes undesirable smell due to the presence of various organic compounds released during its production. Therefore, the aim of this work was to obtain vanillin from lignocellulosic by-products via SSF using selected fungal strains. The process was conducted in a single-use bag bioreactor specially designed for SSF culture on a bench-scale. To test the effect of the raw material and biocatalyst on the sensory properties of the obtained vanillin, the obtained samples were submitted to olfactory evaluation performed by trained panelists.

## 2. Results

### 2.1. Solid-State Fermentation and Isolation of Vanillin

To obtain vanillin, a total of 36 Solid-State Fermentation cultures were performed in the single-use bag bioreactor using 600 g of raw materials. Each of the four strains (*Aspergillus flavus* KKP3556, *Aspergillus* sp. AM31, *Fusarium culmorum* MUT 5855, *Phanerochaete chrysosporium* CBS 246.84) was cultured in triplicate on three different raw materials (brewer’s spent grain (BSG), linseed oil cake (LOC), wheat bran (WB)). After extraction, purification, and crystallization of vanillin, the amounts of vanillin obtained are shown in Table 1 (calculated as mg/kg of d. m. of raw material).

As is shown in Table 1, the highest amount of vanillin was obtained using BSG as a raw material and *P. chrysosporium* CBS246.84 as a biocatalyst (mean 647.3 mg/kg of d. m. of raw material). The second-best result was recorded in SSF with the same strain growing on WB (mean 430.8 mg/kg of d. m. of raw material). BSG proved to be the most suitable raw material because the highest vanillin contents were observed in the post-culture extracts of all strains growing in this by-product. The lowest amounts of vanillin obtained were recorded within all strains when LOC was used as a medium.

As the results show, the type of raw material used in biotechnological processes plays a key role, which results from the qualitative and quantitative composition of individual by-products. Although all raw materials used are excellent sources of lignin, BSG is characterized by the content of a range of nutrients produced during the beer production process (proteins, amino acids, vitamins, minerals), which constitutes a source of valuable nutrients for microorganisms. Pre-treatment of malt—malting and mashing—could also facilitate the microorganisms’ lignin degradation process. WB is also a valuable source of dietary fiber, protein, and other nutrients, including minerals and vitamins. LOC, despite being a rich source of lignin and protein, is characterized by a high content of oil remaining after the pressing process, which could have negatively affected the vanillin biosynthesis process.

Large deviations and lack of perfect repeatability of the result may result from the type of process. Biotechnological processes are difficult to standardize, especially while using organic by-products and whole microbial cells. Ligninolytic activity of the fungi can be unpredictable and is difficult to standardize as it involves the production of various intracellular and extracellular enzymes, as well as intermediary metabolites. It constitutes the part of the organism’s secondary metabolism, determined by a complex set of factors such as nutritional, physiological, and environmental. An additional factor influencing the reproducibility of results is the series of steps leading to vanillin production, including double extraction, purification, and crystallization.

### 2.2. Sensory Value of Vanillin Affected by Various Raw Materials and Biocatalysts

Vanillin has a strong, sweet, warm, and powdery vanilla-like smell. It is often described as creamy, balsamic, and pleasant. Sensory panel results, given in the tables and figures below, showed that synthetic vanillin (SYNTH.) exhibited the least of these features, and the dominant scent note was phenolic. Ethanol extract from the vanilla pods (PODS EXT.) also had a strong phenolic note, but subtle notes typical of vanilla, such as sweet, powdery, and balsamic, were also detected. A commercially purchased extract (COMM.), containing water, sugar, invert sugar, and vanilla extract, particularly showed sweet notes (Table 2).

The above results show that vanillin obtained from agri-food industry by-products such as BSG (CBS_BSG) and WB (CBS_WB), using *Phanerochaete chrysosporium* CBS246.84, is characterized by significantly better aroma qualities compared to synthetic vanillin, as indicated by evaluation of the key note (vanillin-ID) in the samples. The vanillin obtained from BSG exhibited notes similar to the commercially available extract, particularly in terms of vanilla-ID, sweet, and filamentous fungi notes, and has less noticeable chocolate and caramel notes. This sample showed a slightly higher presence of balsamic, creamy, and malty notes. Vanillin obtained from WB was characterized by distinct sweet, chocolate, and creamy notes.

**Figure 1:**
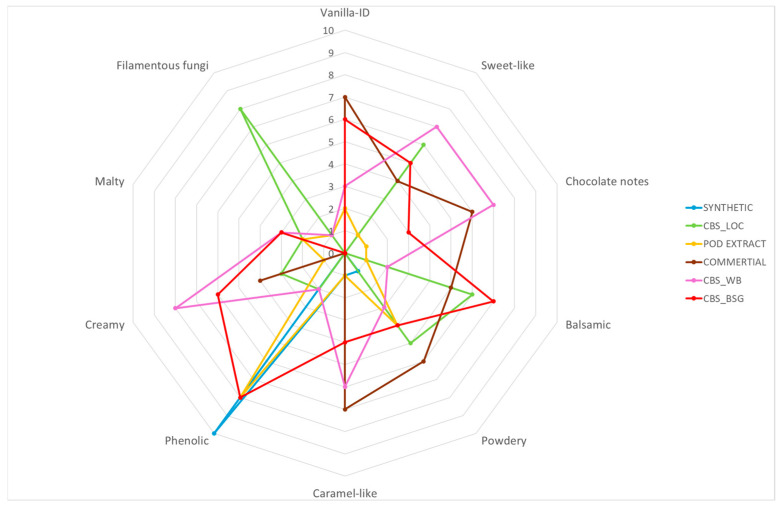
Radar chart of results obtained from the sensory analysis of vanillin obtained from *P. chrysosporium* CBS246.84 cultures and vanillin standards.

Vanillin obtained from LOC (CBS_LOC) proved to be the least attractive raw material among those tested. The compound exhibited no vanilla notes, but panelists indicated the presence of sweet, powdery, and balsamic notes, with a high intensity of filamentous fungi notes (Figure 1).

The results presented in Table 2 show that vanillin obtained from *Aspergillus flavus* KKP3556 culture on WB (KKP_WB) exhibited comparable aroma qualities to commercially purchased vanillin extract. Vanillin obtained from the culture where BSG was used as a substrate (KKP_BSG) also had similar notes; however, panelists indicated the presence of phenolic and malty notes, while powdery and chocolate notes were less noticeable.

**Figure 2:**
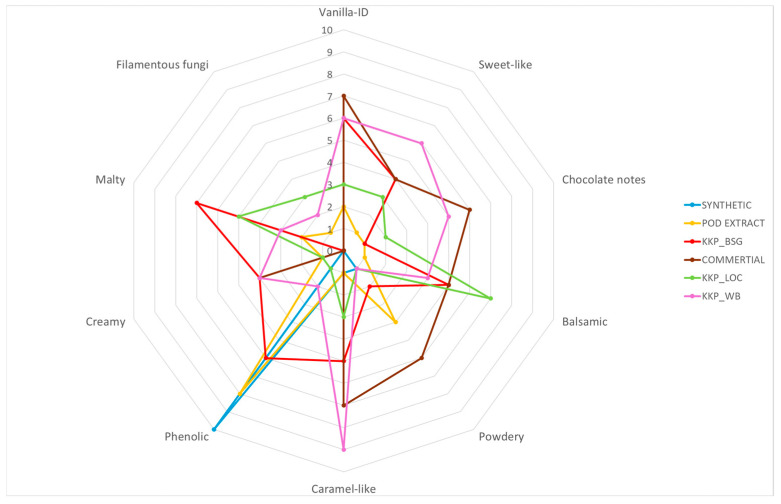
Radar chart of results obtained from the sensory analysis of vanillin obtained from *Aspergillus flavus* KKP3556 cultures and vanillin standards.

Similarly, as in the case of *P. chrysosporium* CBS246.84, vanillin obtained from LOC proved to be the least attractive in comparison to BSG and WB. Nevertheless, the sample showed a more attractive aroma profile compared to alcoholic vanilla pod extract (Figure 2).

Sample MUT_WB (Table 2) was characterized by particularly strong vanilla-ID, sweet-like, and caramel-like notes; additionally, no filamentous fungi note was detected, which makes vanillin obtained from the culture of *Fusarium culmorum* MUT5855 growing on WB an attractive flavor from the consumer’s point of view.

**Figure 3:**
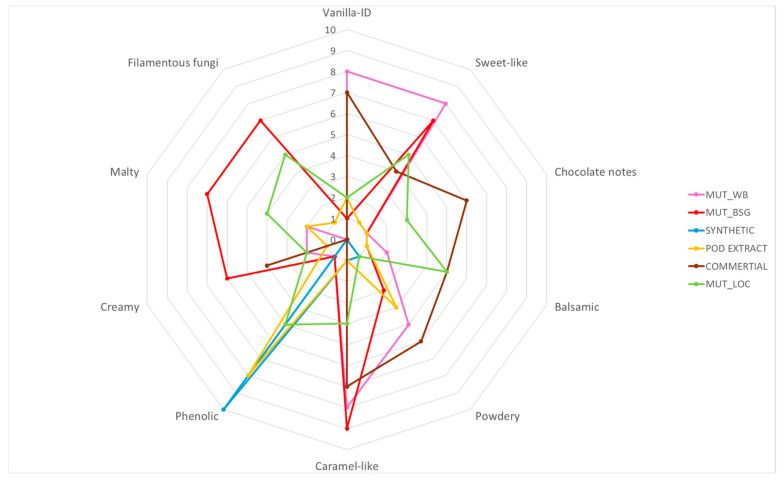
Radar chart of results obtained from the sensory analysis of vanillin obtained from *Fusarium culmorum* MUT5855 cultures and vanillin standards.

As is presented on the radar chart below (Figure 3), samples obtained from fermentation on BSG (MUT_BSG) exhibited particularly strong sweet and caramel-like notes. However, the presence of undesirable malty and filamentous fungi notes were indicated with a barely perceptible note of vanillin.

Vanillin samples obtained with the *Aspergillus* sp. AM31 strain as a biocatalyst do not show attractive scent characteristics, although AM_WB has a distinct vanilla note, and a strong filamentous fungi note is also noticeable (Table 2).

**Figure 4:**
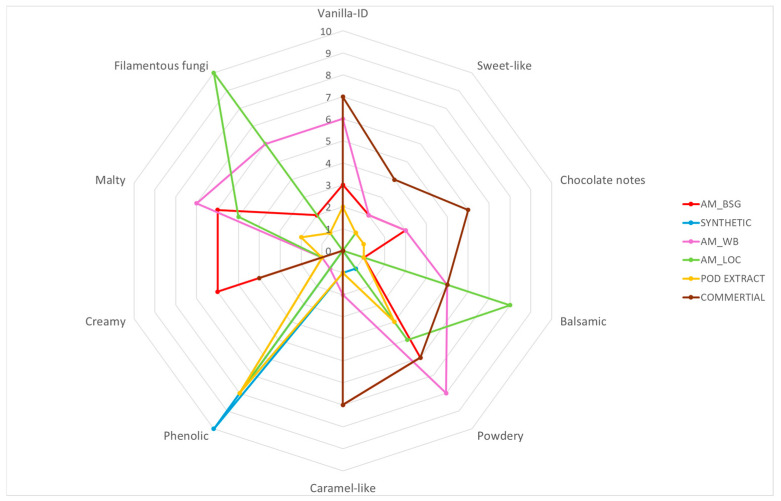
Radar chart of results obtained from the sensory analysis of vanillin obtained from *Aspergillus* sp. AM31 cultures and vanillin standards.

In the AM_LOC sample, a filamentous fungi note is very clearly perceptible; at the same time, panelists assessed the absence of vanilla notes, which is clearly visible in the radar chart presented below (Figure 4).

## 3. Discussion

### 3.1. Solid-State Fermentation and Isolation of Vanillin

The agri-food industry by-products used in this research (brewer’s spent grain, wheat bran, and linseed oil cake) are among the most abundant in Poland. Although their chemical composition may vary depending on the variety, cultivation, and processing method, they are considered the richest sources of lignin. In particular, brewer’s spent grain (BSG) is characterized by a particularly high content (12–28%) compared to wheat bran (WB) (5–6%) [31,32,33]. However, it is worth emphasizing that the main phenolic compound present in the lignin fraction of both of these raw materials is ferulic acid. In BSG extracts, this compound represents >50% of the total polyphenol content (180–300 mg/100 g); in WB, the amount of ferulic acid varies from 20 to 1500 mg/100 g [31,34,35,36]. Although LOC’s main components are lignans and mucilage, research indicates that ferulic acid is one of the major phenolic acids present in this by-product (160–410 mg/100 g), contributing to the overall antioxidant properties [37,38,39]. According to the literature, microorganisms such as white-rot fungi possess the capability to decompose the lignin polymer into smaller aromatic compounds by a complex series of enzymatic reactions. Enzymes such as laccases and peroxidases degrade lignin. Feruloyl esterase is a crucial enzyme that specifically cleaves the ester bonds linking ferulic acid to the hemicellulose and lignin in the plant cell wall, leading it to obtain free ferulic acid. The final phase involves the transformation of the aforementioned compound into vanillin by using an enzyme (or a series of enzymes) that catalyzes a retro-aldol reaction [40].

Studies on the utilization of lignocellulosic by-products primarily concentrate on chemical and microbiological hydrolysis, as well as the extraction of ferulic acid, which is subsequently employed as a substrate for vanillin biosynthesis [7,8,17,19,41]. Another emerging trend in the application of agro-industrial residues involves their incorporation into liquid media as a direct source of vanillin precursors. Chattopadhyay et al. investigated the biosynthesis of vanillin from wheat bran utilizing the *Streptomyces sannanensis* MTCC 6637 strain. The optimal production of vanillin (708 mg/L) was attained after a 5-day incubation period [13].

Solid-State Fermentation (SSF) is a bioprocess that involves the growth of microorganisms on a solid, non-soluble substrate with a low moisture content. The solid material acts as both the physical support and the source of nutrients for the microorganisms [42]. The SSF method has been introduced as a novel approach for the bioconversion of lignocellulosic by-products into vanillin and is receiving attention due to its many efficiencies, no electricity for agitation, and low downstream processing cost as compared to submerge fermentation. Bio-based agricultural by-products have been investigated as alternatives to replace expensive raw material used in microbial vanillin synthesis. The renewability, affordability, accessibility, and sustainable nature of bio-based raw materials provide new insights into natural vanillin production; nevertheless, research on this topic is still in its infancy [43].

The strains used in this study were selected based on the work of Szczepańska et al., where an initial screening of 150 strains was conducted. Of these, seven biocatalysts capable of vanillin biosynthesis from brewer’s spent grains were selected. After optimization using the RSM (Response Surface Methodology) in Erlenmeyer flasks, which took into account factors such as substrate moisture, cultivation temperature, substrate fragmentation degree, and inoculum optical density, the amount of vanillin in post-culture extracts was determined for the strains *Phanerochaete chrysosporium* CBS246.84, *Aspergillus flavus* KKP3556, *Fusarium culmorum* MUT5855, and *Aspergillus* sp. AM31 at levels of 363, 218, 203, and 176 mg/kg d. m. of raw material, respectively. For *P. chrysosporium* CBS246.84, optimization was performed using the RSM (Box–Behnken design) in a single-use bag bioreactor, where three levels of factors such as temperature, time, and airflow were taken into account. This allowed us to achieve a 389% increase in yield, and the amount of vanillin in the post-culture extract was determined as 1413 mg/kg of d. m. of brewer’s spent grains [26]. Based on the results obtained in this study, it can be observed that the use of a single-use bag bioreactor enabled the production of higher vanillin biosynthesis yields compared to cultivation in Erlenmeyer flasks. However, as can be deduced from the results obtained in Table 1 (Section 2.1), there are differences in the amount of isolated vanilla in three repetitions of the same SSF process. Even with the benefits mentioned earlier, SSF also has challenges, including varying fermentation conditions that often lead to inconsistent results (especially in terms of heat distribution) and complications in accurately determining biomass and purification of products, particularly due to the use of heterogeneous organic-growth raw materials, which could lead to the issue related to the repeatability of results [29].

Other approaches to obtain SSF-based vanillin were made among others by Nurika et al., who highlighted the feasibility of utilizing rice straw for vanillin biosynthesis. The experiments were performed using the SSF system in honey jars containing 10 g of rice straw and water inoculated with *Serpula lacrymans*. Additionally, key extraction conditions were identified such as the volume of solvent used and extraction time. After a period of 35 days, a mixture of valuable bio-based compounds was produced, including vanillin at a concentration of 3.957 µg/g of raw material using ethyl acetate as solvent. In comparison, when ethanol was used, the highest yield of vanillin was 2.596 µg/g [27]. Various agricultural lignocellulosic by-products, such as sugarcane bagasse, wheat straw, rice straw, rice bran, and corn cob, were evaluated for their potential biotransformation into vanillin by Mehmood et al. using the SSF technique. The raw materials were placed in an Erlenmeyer flask and inoculated with *Enterobacter hormaechei*, followed by incubation for 48 h at 37.5 °C. Among the agricultural by-products assessed, sugarcane bagasse emerged as the preferred raw material, yielding vanillin in an amount of 0.4760 g/100 g of raw material [44]. Wheat straw was utilized as a source of ferulic acid for vanillin synthesis, employing *Streptomyces sannanensis* and the SSF culture system as investigated by the Mehmood et al. research team. Experiments were carried out in 250 mL Erlenmeyer flasks containing 10 g of wheat straw. The peak production of vanillin (2.74 mg/g) was recorded after 72 h with an incubation temperature of 35 °C [22]. Another example of using the SSF system in order to obtain vanillin is the research conducted by dos Santos et al. Similarly to the previously discussed cases, experiments were conducted in 250 mL Erlenmeyer flasks containing 2 g of green coconut husk and 10 mL of liquid nutrient medium. After inoculation with *Phanerochaete chrysosporium* spore suspension, culture was incubated in 28 °C for 24 h. The post-fermentation medium was extracted with a mixture of methanol and water (60:40 *w*/*w*). As a result, vanillin was obtained in the amount of 52.5 µg/g of raw material [28]. Nisar et al. applied the SSF culture system of *Bacillus cereus* to obtain vanillin. They used 5 g of powdered corn cob in a 250 mL Erlenmeyer flask (incubation time 120 h, moisture content 80%, temperature 30 °C) and achieved the maximum yield of vanillin at 0.104 g/100 g of raw material. Produced vanillin was recovered from post-fermentation medium using the liquid–liquid extraction method by adding water and ethyl acetate. The solvent was evaporated, and the concentrated mixture was dissolved in distilled water and cooled to 4 °C to precipitate crystals of vanillin. HPLC analysis indicated the 99.8% purity of the obtained compound [45].

In this study, the highest amount of vanillin after extraction and purification was obtained when brewer’s spent grain and wheat bran were used as a fermentation medium for fungal strains. Linseed oil cake proved to be the least convenient material when it comes to the amount of biosynthesized and extracted vanillin. The lower efficiency of obtaining vanillin from SSF, where linseed oil cake was used as a medium, probably results from the fact that this raw material is characterized by a high mucilage content. Mucilage is a thick, gel-like substance produced by plants, primarily composed of complex carbohydrates including arabinoxylans, pectin, cellulose, and various other polysaccharide forms [46]. The process of extracting and purification of vanillin is a significant impact of both the type of raw material from which it is extracted and the extraction techniques used for this process. Even in the case of lignin-derived vanillin, the yield is influenced by the source of lignin used for production [5].

### 3.2. Sensory Value of Vanillin Affected by Various Raw Materials and Biocatalysts

The findings indicate that aroma characteristics of vanillin produced through Solid-State Fermentation (SSF) are affected by both the microorganism and the type of raw material utilized in the process. Vanillin derived from the culture of *F. culmorum* MUT5855 cultivated on wheat bran (WB) demonstrated an appealing flavor profile, characterized by notably strong vanilla, sweet-like, and caramel-like notes. Additionally, another preparation that satisfies the sensory criteria for aroma was vanillin extracted from brewer’s spent grain (BSG) and WB using *P. chrysosporium* CBS246.84, which displayed notes similar to those of commercially available extracts. Comparable results were observed for vanillin extracted from the culture of *A. flavus* KKP3556 grown on WB. Vanillin isolated from cultures performed on LOC showed the least attractive odor characteristics compared to vanillin obtained from cultures performed on BSG and WB.

On the basis of the sensory panel, it was concluded that the majority of the biosynthesized vanillin samples had better fragrances than synthetic vanillin, which was characterized by overwhelming phenolic notes. This aspect of sensory evaluation can be attributed to the phenolic nature of the molecule, in which the aromatic ring and hydroxyl group contribute to this characteristic, with a high purity of synthetic form and the lack of other compounds that can affect the reception of the smell.

Extraction and purification of vanillin from natural sources require consideration of several factors, such as the source of vanillin, the effectiveness of the extraction method, the required level of purity, environmental impact, and the associated costs. Moreover, the purification of vanillin, especially from the extract from microbial cultures, is a critical step due to the presence of impurities, including other flavoring compounds and secondary metabolites [5]. A study performed by Lee et al. indicates that the sensory experience of vanilla ice cream products derived from different parts of the world of vanilla beans (Taiwan and Madagascar) primarily diverges in terms of overall aroma [47].

It is worth emphasizing that these are the first studies involving sensory analysis of vanillin obtained by biotechnological methods using the SSF system. The use of a single-use bag bioreactor enabled bench-scale cultivation, ensuring the production of vanillin in quantities sufficient for the sensory panel. Vanillin’s olfactory properties can be distorted by the presence of secondary metabolites produced by microorganisms. Therefore, this type of analysis is crucial, as it determines the attractiveness of the produced compound.

## 4. Materials and Methods

### 4.1. Raw Materials and Chemicals

Ferulic acid and vanillin, used as reference compounds, along with the solvents for extractions and HPLC analysis, were sourced from Sigma-Aldrich (Darmstadt, Germany). Brewer’s spent grain (BSG) was acquired from the brewery located in Wrocław (Złoty Pies, Poland), wheat bran (WB) was purchased from PPHU MILLTEX (Zduny, Poland), and linseed oil cake (LOC) was purchased from MARKET-ROLNICZY (Koźmin Wielkopolski, Poland).

Brewer’s spent grain was subjected to drying at 60 °C for 48 h to eliminate moisture. Once dried, it was processed using an electrical grinder to create a fine powder, which was then sieved through a 0.5 mm mesh. This prepared material served as the raw material for the subsequent optimization experiments. The remaining raw materials (WB and LOC) were purchased in dried and ground form.

### 4.2. Microorganisms

Strain *Aspergillus* sp. AM31 was acquired from the collection of microorganisms at the Department of Food Chemistry and Biocatalysis at Wrocław University of Environmental and Life Sciences (AM) (Wrocław, Poland). *Phanerochaete chrysosporium* CBS246.84 was sourced from the collection of the Westerdijk Fungal Biodiversity Institute (CBS) (Utrecht, The Netherlands). *Aspergillus flavus* KKP3556 was obtained from the Institute of Agricultural and Food Biotechnology State Research Institute Collection of Industrial Microorganisms (KKP) in Warsaw (Poland). *Fusarium culmorum* MUT5855 was procured from the Istituto di Scienze e Tecnologie Chimiche “Giulio Natta”-Consiglio Nazionale delle Ricerche (SCITEC-CNR) in Milan (Italy). All fungal strains were preserved at 4 °C on Czapek’s medium agar slants, which contained the following components per liter: sucrose (30 g), sodium nitrate (3 g), dipotassium phosphate (1 g), magnesium sulfate (0.5 g), potassium chloride (0.5 g), ferrous sulfate (0.001 g), and agar (15 g).

### 4.3. Preparative Scale of Solid-State Fermentation

**Figure 5:**
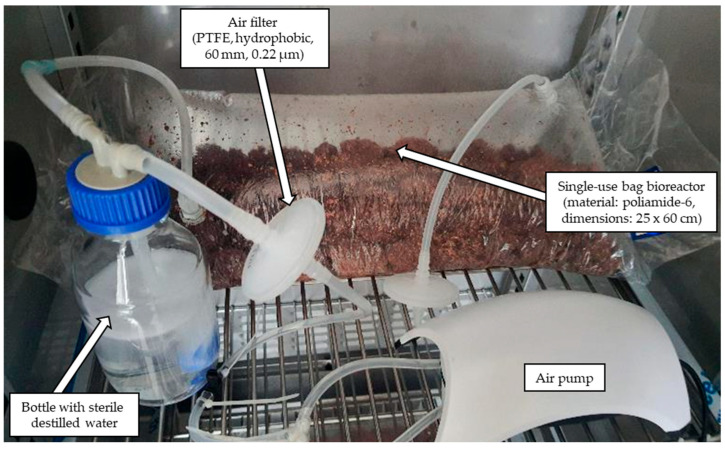
Single-use bag bioreactor used in experiments.

Cultures were performed in a single-use bag bioreactor. The bioreactor chamber (dimensions 25 × 60 cm) was constructed using a polyamide-6 sterilization foil sleeve. Using a heat sealer, 600 g of raw material was sealed inside the chamber, and openings were created for cable glands, into which silicone air supply tubes were inserted (Figure 5). After sterilization (121 °C, 15 min), the moisture content of raw material was adjusted to 60% (*Phanerochaete chrysosporium* CBS246.84, *Fusarium culmorum* MUT5855, *Aspergillus* sp. AM31) and 70% (*Aspergillus flavus* KKP3556) with modified Czapek’s medium (g/L—sucrose 5, sodium nitrate 3, ammonium chloride 1, magnesium sulfate 0.5, potassium chloride 0.5, ferrous sulfate 0.001), containing spores at OD_600_ 0.3 (*Phanerochaete chrysosporium* CBS246.84, *Fusarium culmorum* MUT5855, *Aspergillus* sp. AM31) and 0.2 (*Aspergillus flavus* KKP3556), and incubated for 144 h at temperatures ranging from 25 °C (*Fusarium culmorum* MUT5855, *Aspergillus* sp. AM31, *Aspergillus flavus* KKP3556) to 30 °C (*Phanerochaete chrysosporium* CBS246.84). The strains were selected based on a screening conducted on 150 strains, and the conditions were established during the optimization of the process carried out in Erlenmeyer flasks [26]. To provide oxygen to the bioreactor, air sterilized by filtration (PTFE hydrophobic filters 60 mm, 0.22 µm) was introduced by aquarium air pumps through a silicone tube. Air-flow rate was measured at the outlet of the bioreactor using a rotameter (Kobold, model no. KFR-2114N0, Arnhem, The Netherlands); to adjust the air flow (1.75 NL/min), a choke valve was mounted on the air-flow line. To prevent the solid substrate from drying out, the air was humidified by passing it through a bottle containing sterile distilled water.

### 4.4. Extraction and Purification

The post-fermentation medium was extracted three times on a shaker for 12 h with 95% ethanol (1:4 *w*/*w* ratio of medium to solvent). The ethanol was then evaporated in a vacuum evaporator to the remaining 200 mL, and 800 mL of ethyl acetate was added, followed by extraction on a shaker for 12 h. After separation of the two solvents, the ethyl acetate fraction was concentrated under reduced pressure and purified by silica gel column chromatography (Sigma-Aldrich, Darmstadt, Germany) using ethyl acetate as the eluent. The collected fraction containing vanillin was evaporated, and the compound was crystallized using hexane. The codes of individual samples are presented in Table 3.

### 4.5. Analysis Procedure

The analysis was conducted via HPLC with an UltiMate 3000 instrument (Dionex, Sunnyvale, CA, USA) equipped with a UV detector and Luna 5u C18 packed column (25 cm × 4.6 mm, 5 μm, Phenomenex, Torrance, CA, USA). The mobile phase was composed of aqueous 0.5% formic acid (solution A) and methanol (solution B) with a flow rate of 1 mL/min. The gradient programming was set as follows: A/B (*v*/*v*): 70:30 (0–3 min), 25:75 to 11 min, 0:100 to 13 min, and then returning to 70:30 at 21 min. The absorbance was recorded at both 281 nm and 254 nm. For the quantitative determination of vanillin, a standard curve was created, allowing for the calculation of its content in kg of dry matter from the by-product. 

### 4.6. Sensory Analysis

The obtained vanillin was subjected to sensory analysis at the Sensory Analysis Laboratory of the Wrocław University of Environmental and Life Sciences, according to recognized methodology [48,49]. Six panelists (aged 24–32) who follow a strict training regime, including regular two-hour training sessions to assess olfactory sensitivity and the ability to discriminate between odors, evaluated the samples. The panelists were familiarized with the lexicon (Supplementary Materials: Lexicon); all concepts and terms were explained to them, and the values assigned to the reference materials were verified. Prior to the sensory evaluation of the samples, a training session was conducted using the reference materials included in the lexicon to calibrate the scales. The samples, coded with 3-digit numbers (20 mL covered glass probes containing 0.5% of purified vanillin dissolved in ultrapure anhydrous ethanol), were delivered to the panelists in random order. Sample evaluation was performed in a tasting room with individual booths in which both the light (70–90 fc) and temperature (22 ± 1 °C) were monitored and controlled. As a references, synthetic vanillin, a commercially available vanilla extract solution (Royal Brand^®^), and ethanol extract from vanilla pods were used. Additionally, extracts obtained from raw materials (brewer’s spent grain, linseed oil cake, wheat bran) were prepared for the sensory panel. A group of panelists assessed the vanilla-ID, sweet-like, chocolate, balsamic, powdery, caramel-like, phenolic, creamy, malty, and filamentous fungi notes in the samples on a scale of 0 (no intensity) to 10 (extremely strong intensity). The full lexicon is available in the Supplementary Materials (Table S1).

### 4.7. Statystical Analysis

Statistica 13.3 (StatSoft, Kraków, Poland) software was used for data analysis. The data were subjected to Tukey’s Honest Significant Test (Tukey’s HSD).

## 5. Conclusions

Vanillin is one of the most desirable flavoring agents in the food and pharmaceutical industries, giving products a desirable taste and smell. The market is dominated by chemically obtained vanillin. Consumers are increasingly demanding natural ingredients over synthetic ones, driven by a heightened awareness of health and wellness. In this work, vanillin was biosynthesized via SSF in a single-use bag bioreactor using selected fungal strains and lignocellulosic by-products such as brewer’s spent grain (BSG), wheat bran (WB), and linseed oil cake (LOC). The highest amount of vanillin was obtained using BSG as a raw material and *P. chrysosporium* CBS246.84 as a biocatalyst (mean 647.3 mg/kg of d. m. of raw material). The second-best result was recorded in SSF with the same strain growing on WB (mean 430.8 mg/kg of d. m. of raw material). Using a sensory panel, the fragrance properties of the obtained compound were assessed and compared with synthetic vanillin and its natural extracts. The results show that the aroma characteristics of vanillin obtained by SSF are influenced by both the microorganism and the type of raw material used in the process. Vanillin obtained from the cultures of *F. culmorum* MUT5855 growing on WB and *P. chrysosporium* CBS246.84 on BSG and WB proved to be attractive flavors as they were characterized by notes similar to the commercially available extract. These are the first studies involving sensory analysis of biotechnologically obtained vanillin using the SSF system.

## Figures and Tables

**Table 1:** The amount of vanillin (mg/kg of d. m. of raw material) obtained after purification from Solid-State Fermentation.

Microorganism	Rep.	BSG ^1^	SD ^4^	WB ^2^	SD ^4^	LOC ^3^	SD ^4^
*Aspergillus flavus* KKP3556	I	402.4	±7.9	140.3	±16.1	158.2	±11.3
	II	387.3		160.4		180.7	
	III	399.2		172.2		172.2	
*Aspergillus* sp. AM31	I	572.5	±231.7	203.0	±42.6	116.5	±74.4
	II	136.2		244.3		256.4	
	III	218.8		159.0		230.7	
*Fusarium culmorum* MUT5855	I	279.9	±36.3	149.8	±15.5	154.4	±25.6
	II	285.5		160.1		170.0	
	III	220.0		180.4		120.0	
*Phanerochaete chrysosporium* CBS246.84	I	782.3	±127.3	481.5	±46.3	190.2	±30.6
	II	529.3		390.5		150.2	
	III	630.2		420.3		210.3	

^1^ Brewer’s spent grain. ^2^ Wheat bran. ^3^ Linseed oil cake. ^4^ Standard deviation.

**Table 2:** The modal mean of results obtained from the sensory analysis of vanillin obtained from *P. chrysosporium* CBS246.84, *A. flavus* KKP3556, *F. culmorum* MUT5855, and *Aspergillus* sp. AM31 cultures and vanillin standards.

Sample	Vanilla-ID	Sweet-Like	Chocolate	Balsamic	Powdery	Caramel-Like	Phenolic	Creamy	Malty	Filamentous Fungi
SYNTH.	0 ^c^	0 ^c^	0 ^c^	0 ^c^	1 ^ac^	1 ^c^	10 ^a^	0 ^c^	0 ^c^	0 ^b^
PODS EXT.	2 ^b^	1 ^c^	1 ^c^	1 ^c^	4 ^b^	1 ^c^	8 ^a^	1 ^c^	2 ^ab^	1 ^b^
COMM.	7 ^a^	4 ^b^	6 ^a^	5 ^b^	6 ^a^	7 ^a^	0 ^c^	4 ^b^	0 ^c^	0 ^b^
CBS_BSG	6 ^a^	5 ^ab^	3 ^b^	7 ^a^	4 ^b^	4 ^b^	8 ^a^	6 ^ab^	3 ^a^	0 ^b^
CBS_WB	3 ^b^	7 ^a^	7 ^a^	2 ^c^	3 ^bc^	6 ^a^	2 ^b^	8 ^a^	3 ^a^	1 ^b^
CBS_LOC	0 ^c^	6 ^a^	0 ^c^	6 ^a^	5 ^ab^	0 ^c^	2 ^b^	3 ^b^	2 ^ab^	8 ^a^
KKP_BSG	6 ^a^	4 ^ab^	1 ^b^	5 ^b^	2 ^c^	5 ^b^	6 ^b^	4 ^a^	7 ^a^	0 ^c^
KKP_WB	6 ^a^	6 ^a^	5 ^a^	4 ^b^	1 ^c^	9 ^a^	2 ^c^	4 ^a^	3 ^c^	2 ^ab^
KKP_LOC	3 ^b^	3 ^b^	2 ^b^	7 ^a^	1 ^c^	3 ^bc^	1 ^c^	1 ^b^	5 ^b^	3 ^a^
MUT_BSG	1 ^b^	7 ^a^	1 ^c^	1 ^c^	3 ^c^	9 ^a^	1 ^c^	6 ^a^	7 ^a^	7 ^a^
MUT_WB	8 ^a^	8 ^a^	1 ^c^	2 ^b^	5 ^ab^	8 ^a^	1 ^c^	2 ^b^	2 ^bc^	0 ^c^
MUT_LOC	2 ^b^	5 ^b^	3 ^b^	5 ^a^	1 ^c^	4 ^c^	5 ^b^	2 ^b^	4 ^b^	5 ^b^
AM_BSG	3 ^b^	2 ^b^	3 ^b^	1 ^c^	6 ^ab^	7 ^a^	0 ^c^	6 ^a^	6 ^ab^	2 ^c^
AM_WB	6 ^a^	2 ^b^	3 ^b^	5 ^b^	8 ^a^	2 ^bc^	1 ^c^	1 ^c^	7 ^a^	6 ^b^
AM_LOC	0 ^c^	1 ^c^	0 ^c^	8 ^a^	5 ^b^	0 ^c^	8 ^a^	1 ^c^	5 ^b^	10 ^a^

Values, within the column, followed by the same letters are not statistically different in Tukey’s HSD test.

**Table 3:** Sample codes of isolated vanillin solutions.

Code	Microorganism	Raw Material
KKP_BSG	*Aspergillus flavus* KKP3556	Brewer’s spent grain
KKP_WB		Wheat bran
KKP_LOC		Linseed oil cake
AM_BSG	*Aspergillus* sp. AM31	Brewer’s spent grain
AM_WB		Wheat bran
AM LOC		Linseed oil cake
MUT_BSG	*Fusarium culmorum* MUT5855	Brewer’s spent grain
MUT_WB		Wheat bran
MUT_LOC		Linseed oil cake
CBS_BSG	*Phanerochaete chrysosporium* CBS246.84	Brewer’s spent grain
CBS_WB		Wheat bran
CBS_LOC		Linseed oil cake

## Data Availability

The data are contained within the article; further inquiries can be directed to the corresponding authors.

## Supplementary Materials

The following supporting information can be downloaded at: https://www.mdpi.com/article/10.3390/molecules30204109/s1, Table S1: Lexicon for sensory descriptive analysis.
