# Supplementary material for: Sensory Evaluation of Vanillin Obtained by Fungi in the Solid-State Fermentation from Agri-Food Industry By-Products
Source: Molecules. 2025 Oct 16;30(20):4109. doi: 10.3390/molecules30204109 (PMC12566194; doi:10.3390/molecules30204109)
Supplement: Supplementary file 1 [file molecules-30-04109-s001.zip › molecules-3859958-supplementary.pdf]

Table S1. Lexicon for sensory descriptive analysis.

Smell attribute	Definition	Reference materials
Vanilla-ID	A sweet scent, characteristic of a cut vanilla pod	Split vanilla pod - 10<br>Vanilla ice cream - 5
Sweet	A hardly perceptible scent, reminiscent of sugar syrup or honey with a delicate fragrance profile	Honey with a mild fragrance profile - 10<br>20% sugar solution - 3
Cholotate-like	The scent of milk chocolate, with delicate notes of cocoa	Milk chocolate - 8<br>Instant cocoa in milk - 4
Balsamic	A rich and full-bodied scent with woody and resinous notes; pleasant and relaxing	Resinous essential oil - 9<br>Ice cream stick - 4
Powdery	A clean and mild scent that causes the sensation of drying the nasal mucosa	Baby talcum powder - 10<br>Wheat flour - 3
Caramel-like	A sweet scent with notes of toffee and burnt sugar	Cane sugar - 4<br>Caramel syrup - 10
Phenolic	An irritating odor reminiscent of medicinal substances, such as antiseptics. May contain smoky notes	10% isopropanol solution - 5
Creamy	A delicate and mild scent, often associated with dairy products. A full-bodied aroma, characteristic of products with a high fat content	Butter - 10<br>Cream 30% - 4

Off-flavours	Definition
Malty	A cereal scent with notes typical of wet cardboard.
Filamentous fungi	Odor typical of liquid or plate cultures of filamentous fungi. May be described as the odor of a damp room
